# Supplementary material for: A novel nutritional supplement containing amino acids and chromium decreases postprandial glucose response in a randomized, double-blind, placebo-controlled study
Source: PLoS One. 2020 Jun 24;15(6):e0234237. doi: 10.1371/journal.pone.0234237 (PMC7313729; doi:10.1371/journal.pone.0234237)
Supplement: S1 Table — (PDF) [file pone.0234237.s001.pdf]

S1 Table. Inclusion and exclusion criteria

Inclusion Criteria
Male or female 18 to 50 years of age
BMI 25-29.9 (±0.5) kg/m²
Female participant is not of child bearing potential, which is defined as females who have had a hysterectomy or oophorectomy, bilateral tubal ligation or are post-menopausal (natural or surgically with > 1 year since last menstruation)
OR
Females of childbearing potential who agree to use a medically approved method of birth control and have a negative urine pregnancy test result. Acceptable methods of birth control include:
Hormonal contraceptives including oral contraceptives, hormone birth control patch (Ortho Evra), vaginal contraceptive ring (NuvaRing), injectable contraceptives (Depo-Provera, Lunelle), or hormone implant (Norplant System)
Double-barrier method
Intrauterine devices
Non-heterosexual lifestyle or agrees to use contraception if planning on changing to heterosexual partner(s)
Vasectomy of partner (shown successful as per appropriate follow-up)
White North American or African American
Stable body weight defined as no more than ± 3 kg change during the last 2 months
Agree to maintain consistent dietary habits and physical activity levels for the duration of the study
Self-perceived general good health as per the general health questionnaire

Fasting blood glucose < 6.1 mmol/L at screening
Healthy as determined by laboratory results and medical history
Willingness to complete questionnaires, records, and diaries associated with the study and to complete all clinic visits<br><br>Has given voluntary, written, informed consent to participate in the study
**Exclusion Criteria**
Women who are pregnant, breast feeding, or planning to become pregnant during the trial
Any medical condition(s) or medication(s) known to significantly affect glucose metabolism. Significance to be assessed by the Qualified Investigator
Has undergone procedures that requires cleansing of the bowel, such as colonoscopy or barium enema within three months prior to randomization
Type I or Type II diabetes
Use of over-the-counter medication or natural health products that affect glucose metabolism is prohibited within 2 weeks of enrollment and during this study
Use of anti-biotics within 2 weeks of enrollment
Use of probiotic supplements within 2 weeks of enrollment
Use of cholesterol lowering medications
Use of blood pressure medications
Use of over-the-counter decongestants that contain ephedrine or pseudoephedrine within 2 weeks of enrollment
Use of acute or over the counter medications within 72 h of test product consumption
Use of Tricyclic antidepressants or any other medication that will modify bowel function

Metabolic diseases and chronic gastrointestinal diseases (IBS, Crohns etc.)
Allergy to test product or placebo ingredients
Participants restricted to a vegetarian or vegan diet
Intolerance to lactose or gluten
Irregular dietary habits, including: intermittent fasting, regularly skipped meals, and individuals who do not typically eat breakfast.
Any form of acute infection within 2 weeks of enrollment
Individuals who are immuno-compromised (HIV positive, on anti-rejection medication, rheumatoid arthritis)
History of gastrointestinal dysfunction or surgery that may influence digestion or absorption
History of blood/bleeding disorders
Individuals who are averse to venous catheterization or capillary blood sampling
Current diagnosis of cancer, except skin cancers completely excised with no chemotherapy or radiation with a follow up that is negative. Volunteers with cancer in full remission for more than five years after diagnosis are acceptable
Individuals who have planned surgery during the course of the study
Alcohol or drug abuse within the last 6 months
Currently active smokers (tobacco products, and e-cigarettes) or smoking within the 6 months of enrollment
Blood or plasma donation in the past 2 months
Participants planning to donate blood during, or within 30 days following completion of the study
Use of medical marijuana

History of, or current, psychiatric disease
Unstable medical conditions as determined by QI
Clinically significant abnormal laboratory results at screening
Participation in a clinical research trial within 30 days prior to randomization
Individuals who are cognitively impaired and/or who are unable to give informed consent
Any other condition which in the Qualified Investigator's opinion may adversely affect the individual's ability to complete the study or its measures or which may pose significant risk to the individual
Medical or psychological condition that in the Qualified Investigator’s opinion could interfere with study participation
