# Supplementary material for: A novel nutritional supplement containing amino acids and chromium decreases postprandial glucose response in a randomized, double-blind, placebo-controlled study
Source: PLoS One. 2020 Jun 24;15(6):e0234237. doi: 10.1371/journal.pone.0234237 (PMC7313729; doi:10.1371/journal.pone.0234237)
Supplement: S1 File — (PDF) [file pone.0234237.s004.pdf]

CLINICAL PROTOCOL COVER PAGE

**Protocol Title:**	A multi-centre randomized, double-blind, placebo-controlled, crossover, proof-of-concept study to evaluate the efficacy and safety of Good Idea^{TM} on glucose homeostasis in a healthy population
Protocol Number:	17GGHD
**Protocol Date:**	December 09, 2016
Amendment(s):	
**Study Phase:**	Phase I
**Study Design:**	Multi-centre, randomized, double-blind, placebo-controlled, crossover, proof-of-concept
**Sponsor:**	Double Good AB<br>Scheelevägen 22<br>223 63 Lund, Sweden
**Sponsor Contact:**	Elin Östman<br>Scheelevägen 22<br>223 63 Lund, Sweden<br>+467-0682-3010<br>elin.ostman@goodideadrinks.com
**CRO:**	KGK Synergize Inc.<br>Suite 1440, One London Place<br>255 Queens Ave<br>London Ontario N6A 5R8<br>Canada<br>519-438-9374
**Qualified Investigator:**	Tetyana Pelipyagina, MD<br>KGK Synergize Inc.<br>519-438-9374<br><br>Nicole Craven, MD.<br>KGK USA, Orlando Florida Site<br>407-674-7311

PROTOCOL SIGNATURE SHEET

Name	Signature	Date
Sponsor:<br>Elin Östman<br>Scheelevägen 22<br>223 63 Lund, Sweden<br>+467-0682-3010<br>elin.ostman@goodideadrinks.com		
Scientific Director:<br>Malkanthi Evans, PhD<br>Scientific Director<br>KGK Synergize Inc.<br>255 Queens Ave.,<br>Suite 1440<br>London, ON N6A 5R8<br>Canada		

1 List of Abbreviations and Symbols

ADE	adverse device effect
AE	adverse event
ALT	alanine transaminase
AST	aspartate aminotransferase
BMI	body mass index
CBC	complete blood count
Cl	chloride
cm	centimetre
CRF	case report form
EC	ethics committee
EDTA	diaminoethanetetraacetic acid
e.g.	for example
et al	and others
g	gram
GCP	Good Clinical Practice
GGT	gamma-glutamyltransferase
Ibs	pounds
ICH	International Conference of Harmonization
IEC	Independent Ethics Committee
IRB	Institutional Review Board
K	potassium
kg	kilogram
L	liter
m	meter
mg	milligram
ml	milliliter
Na	Sodium
RBC	red blood cell count
RDW	red cell distribution width
SAE	serious adverse event
SOP	standard operating procedure
SST	serum separating tube
TPD	Therapeutic Products Directorate
ULN	upper limit of normal
WBC	white blood cell count

TABLE OF CONTENTS

1 LIST OF ABBREVIATIONS AND SYMBOLS 3
2 INTRODUCTION 6
3 STUDY OBJECTIVES 8
4 STUDY DESIGN 9
5 SELECTION OF STUDY POPULATION 9
5.1 INCLUSION CRITERIA 10
5.2 EXCLUSION CRITERIA 10
5.3 CONCOMITANT MEDICATIONS 12
5.4 EARLY WITHDRAWAL 12
6 INVESTIGATIONAL PRODUCT 13
6.1 MANUFACTURING AND STORAGE 13
6.2 LABELING AND CODING 14
6.3 INVESTIGATIONAL PRODUCT (GOOD IDEA™) 14
6.4 PLACEBO: 15
6.5 DIRECTIONS TO BE FOLLOWED PRIOR TO BASELINE AND VISIT 3: 15
6.6 RANDOMIZATION 17
***6.7* UNBLINDING AND ALLOCATION CONCEALMENT** 17
7 STUDY ASSESSMENTS 17
7.1 SCREENING (DAY -28 TO DAY -1; VISIT 1) 17
7.2 DAY 0 – BASELINE (VISIT 2) 18
7.3 WASHOUT PERIOD (7 DAYS) 19
7.4 DAY 8 (+6; VISIT 3) 20
7.5 FOLLOW-UP PHONE CALL (DAY 14) ..... FEL! BOKMÄRKET ÄR INTE DEFINIERAT.
7.6 CLINICAL ASSESSMENTS AND PROCEDURES 20
7.6.1 Height, Weight *21*
7.6.2 Blood Pressure *21*
7.6.3 Compliance *21*
7.7 LABORATORY ANALYSES 21
7.8 TERMINATION OF THE TRIAL 22
7.9 PROTOCOL AMENDMENTS 22
8 SAFETY INSTRUCTIONS AND GUIDANCE 23
8.1 ADVERSE EVENTS AND LABORATORY ABNORMALITIES 23

8.1.1 Adverse Events *23*
8.1.2 Serious Adverse Event *24*
8.1.3 Unexpected Adverse Reaction *24*
8.1.4 Laboratory Test Abnormalities *24*
8.2 Treatment and Follow-up of AEs and Laboratory Abnormalities **25**
8.2.1 Treatment and Follow-up of AEs *25*
8.2.2 Treatment and Follow-up of Laboratory Abnormalities *25*
8.3 Reporting of SAEs and Unexpected Adverse Reactions **25**
9 STATISTICAL EVALUATION **26**
9.1 Determination of sample size **26**
9.2 Analysis Population **26**
9.3 Analysis Plan **26**
9.3.1 Premature Discontinuation Description *27*
9.3.2 Safety *27*
9.3.3 Premature Discontinuation Description .... ***Fel! Bokmärket är inte definierat.***
9.3.4 Safety .... ***Fel! Bokmärket är inte definierat.***
9.4 Protocol Deviation Description **27**
9.5 Protocol Amendments **27**
10 DATA COLLECTION AND STORAGE **27**
11 ETHICAL ASPECTS OF THE STUDY **28**
11.1 IRB Approval **28**
11.2 Volunteer Information and Informed Consent **28**
11.3 Potential Risks and Procedures to Minimize Risk **28**
12 QUALITY ASSURANCE AND QUALITY CONTROL **29**
12.1 Auditing **29**
12.2 Monitoring **29**
12.3 Data Management **29**
13 APPENDICES **32**
13.1 Appendix 1 Schedule of Assessments **32**
13.2 Appendix 2: List of Over-the-counter Medication or Natural Health Product that Affect Glucose Metabolism .... Fel! Bokmärket är inte definierat.

2 INTRODUCTION

The balance between the rate of blood glucose appearance and its clearance after consumption of a food defines post-prandial blood glucose homeostasis [1]. Sharp increases in post-prandial blood glucose or insulin, termed a 'spike', have been associated with impairments with insulin sensitivity, in some cases leading to insulin resistance and related disorders [2]. A food's relative impact on post-prandial blood glucose is defined as its glycemic index (GI). GI varies across food categories and is impacted by modern high temperature, high pressure processing techniques [3]. Due to the latter, an increasing number of foods available in today's commercial food market are classified as high in GI [3]. In many cases making it hard for the general consumer to avoid blood glucose spikes associated with consumption of these foods. Therefore, a need exists for natural interventions that can aid in the maintenance of glucose homeostasis in healthy adults.

A rapidly digested protein derived from milk, whey, has been shown to have potent insulinogenic effects when administered to healthy adults [4]. Previous work has attributed much of this effect to post-prandial increases in certain amino acids contained in whey protein, specifically, lysine, threonine, and the branch chain amino acids leucine, isoleucine, and valine [5]. Evidence suggests that intake of whey protein or a mixture of these five amino acids in conjunction with a carbohydrate rich meal has beneficial effects on the post-prandial rise in blood glucose through a 47% reduction in the incremental area under the curve [5]. Moreover, this change in post-prandial blood glucose was not accompanied by increased insulin, indicating an insulin independent effect that may be due to increased insulin sensitivity [5]. The mechanism of action eliciting this effect remains to be answered but may include actions on B-cells and earlier promotion of insulin release post-prandially [4;5].

Chromium is an essential mineral that has been investigated for its effects on post-prandial blood glucose and long-term control of glucose homeostasis. It is proposed that chromium positively effects blood glucose by potentiating the action of insulin signaling in the cell to enhance carbohydrate metabolism [6]. The effect of chromium on post-prandial blood glucose homeostasis has been investigated in human clinical trials. A chromium and L-arabinose mixture immediately following an acute oral sucrose challenge was found to significantly and consistently lowered post-prandial blood glucose when tested repeatedly [2]. Moreover, this improvement in post-prandial blood glucose was accompanied by a significant decrease in insulin levels from baseline, indicating this effect was insulin independent and may be due to an increase in insulin sensitivity. A clinical trial comparing the effect of two different doses of chromium on post-prandial glucose found that both a 400ug and 800ug dose significantly reduced blood glucose after a carbohydrate rich meal, indicating that doses above this threshold do not infer more benefit on glucose homeostasis [6].

Therefore, this study aimed to investigate the effect of a formula containing chromium picolinate and the five amino acids lysine, threonine, leucine, isoleucine, valine, called Good Idea™, on post-prandial blood glucose homeostasis after a standard test meal given to healthy adults.

3 STUDY OBJECTIVES

The objective of this study is to investigate the effect of Good Idea™ on glucose homeostasis in a healthy population.

Primary outcome is the difference of the two-hour iAUC $_{(0-120\ min)}$ for intravenous blood **glucose** between Good Idea™ and the placebo following a standardized meal.

Secondary outcomes:

1. The difference of the two-hour iAUC $_{(0-120\ min)}$ for capillary blood **glucose** between Good Idea™ and the placebo following a standardized meal.
2. The difference in the two-hour C_{max}, and $T_{max\ (0-120\ min)}$ of capillary **glucose** between Good Idea™ and the placebo following a standardized meal.
3. The difference in the two-hour iAUC $_{(0-120\ min)}$ intravenous **insulin** iAUC between Good Idea™ and the placebo following a standardized meal.
4. The difference in the two-hour C_{max} and $T_{max\ (0-120\ min)}$ of intravenous **glucose** and **insulin** between Good Idea™ and the placebo following a standardized meal.
5. The difference in the three-hour iAUC $_{(0-180\ min)}$ for intravenous blood **glucose** and **insulin** between Good Idea™ and the placebo following a standardized meal.
6. The difference in the three-hour C_{max} and $T_{max\ (0-180\ min)}$ of intravenous **glucose** and **insulin** between Good Idea™ and the placebo following a standardized meal.
7. The difference in the three-hour iAUC $_{(0-180\ min)}$, C_{max}, and T_{max} of capillary **glucose** between Good Idea™ and the placebo following a standardized meal.
8. Eating patterns as assessed by a 3-day food record for the weeks prior to days 0 and 8

Safety outcomes:

1. The effects of supplementation with Good Idea™ on vital signs (blood pressure and heart rate) compared to placebo
2. The change in Hematology from screening to end-of-study as assessed by: hemoglobin, hematocrit, platelet count, red blood cell count (RBC), red cell indices, red cell distribution width (RDW), white blood cell count (WBC) and differentials (neutrophils, lymphocytes, monocytes, eosinophils, basophils) between Good Idea™ and the placebo at screening and on day 8
3. The change in Clinical chemistry from screening to end-of-study as assessed by alanine aminotransferase (ALT), aspartate aminotransferase (AST), bilirubin, creatinine, electrolytes (Na, K, Cl), and between Good Idea™ and the placebo
4. Incidence of adverse events in the Good Idea™ and placebo group over the course of the study

4 STUDY DESIGN

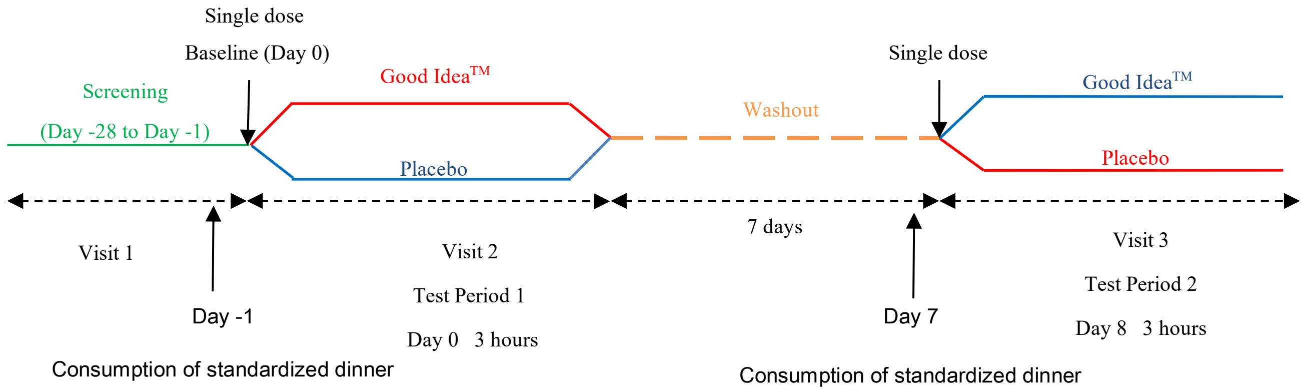

This will be a multi-center, randomized, double-blind, placebo-controlled, crossover, proof-of-concept study to evaluate the efficacy and safety of Good Idea™ on glucose homeostasis. To determine the primary and secondary objectives, assessments will be conducted at screening and on days 0 and 8.

With an estimated 20% attrition over the course of this study, the planned sample size is sixty participants: 30 White North Americans (includes Hispanic, non-Hispanic, Aboriginal and Asian) and 30 African American. Both groups would be required to have an equal number of males and females enrolled and randomized to the 2 study arms in a double-blind manner at a ratio of 1:1.

Study Arm	Number of Participants
Good Idea™ → Placebo	N = 30
Placebo → Good Idea™	N = 30
**Total**	**N = 60**

To evaluate primary, secondary, and safety outcomes, study assessments will be conducted at baseline and all study visits.

The study will be conducted at the KGK clinic sites in London ON, and Orlando FL.

5 SELECTION OF STUDY POPULATION

This study will include 60 healthy male and female participants. Each participant will have to fulfill the inclusion criteria and not meet any of the exclusion criteria as described in sections 5.1 and 5.2.

5.1 Inclusion Criteria

1. Male or female 18 to 50 years of age
2. BMI 25-29.9 (±0.5) kg/m^2
3. Female participant is not of child bearing potential, which is defined as females who have had a hysterectomy or oophorectomy, bilateral tubal ligation or are post-menopausal (natural or surgically with > 1 year since last menstruation)

   OR

   Females of childbearing potential who agree to use a medically approved method of birth control and have a negative urine pregnancy test result. Acceptable methods of birth control include:

   - Hormonal contraceptives including oral contraceptives, hormone birth control patch (Ortho Evra), vaginal contraceptive ring (NuvaRing), injectable contraceptives (Depo-Provera, Lunelle), or hormone implant (Norplant System)
   - Double-barrier method
   - Intrauterine devices
   - Non-heterosexual lifestyle or agrees to use contraception if planning on changing to heterosexual partner(s)
   - Vasectomy of partner (shown successful as per appropriate follow-up)
4. White North American (should include Hispanic, non-Hispanic, Aboriginal and Asian) or African American
5. Stable body weight defined as no more than ± 3 kg change during the last 2 months
6. Agree to maintain consistent dietary habits and physical activity levels for the duration of the study
7. Self-perceived general good health as per the general health questionnaire (Appendix 2: Self-Rated Heath Item)
8. Fasting blood glucose < 6.1 mmol/L at screening
9. Healthy as determined by laboratory results and medical history
10. Willingness to complete questionnaires, records, and diaries associated with the study and to complete all clinic visits
11. Has given voluntary, written, informed consent to participate in the study

5.2 Exclusion Criteria

1. Women who are pregnant, breast feeding, or planning to become pregnant during the trial

2. Any medical condition(s) or medication(s) known to significantly affect glucose metabolism. Significance to be assessed by the Qualified Investigator
3. Has undergone procedures that requires cleansing of the bowel, such as colonoscopy or barium enema within three months prior to randomization
4. Type I or Type II diabetes
5. Use of over-the-counter medication or natural health products that affect glucose metabolism is prohibited within 2 weeks of enrollment and during this study (please refer to section 5.3 Concomitant Medications)
6. Use of anti-biotics within 2 weeks of enrollment
7. Use of probiotic supplements within 2 weeks of enrollment
8. Use of cholesterol lowering medications
9. Use of blood pressure medications
10. Use of over-the-counter decongestants that contain ephedrine or pseudoephedrine within 2 weeks of enrollment (please refer to 5.3 Concomitant Medications)
11. Use of acute or over the counter medications within 72 h of test product consumption
12. Use of Tricyclic antidepressants or any other medication that will modify bowel function
13. Metabolic diseases and chronic gastrointestinal diseases (IBS, Crohns etc.)
14. Allergy to test product or placebo ingredients
15. Participants restricted to a vegetarian or vegan diet
16. Intolerance to lactose or gluten
17. Irregular dietary habits, including: intermittent fasting, regularly skipped meals, and individuals who do not typically eat breakfast.
18. Any form of acute infection within 2 weeks of enrollment
19. Individuals who are immuno-compromised (HIV positive, on anti-rejection medication, rheumatoid arthritis)
20. History of gastrointestinal dysfunction or surgery that may influence digestion or absorption
21. History of blood/bleeding disorders
22. Individuals who are averse to venous catheterization or capillary blood sampling
23. Current diagnosis of cancer, except skin cancers completely excised with no chemotherapy or radiation with a follow up that is negative. Volunteers with cancer in full remission for more than five years after diagnosis are acceptable
24. Individuals who have planned surgery during the course of the study
25. Alcohol or drug abuse within the last 6 months
26. Currently active smokers (tobacco products, and e-cigarettes) or smoking within the 6 months of enrollment
27. Blood or plasma donation in the past 2 months

28. Participants planning to donate blood during, or within 30 days following completion of, the study
29. Use of medical marijuana
30. History of, or current, psychiatric disease
31. Unstable medical conditions as determined by QI
32. Clinically significant abnormal laboratory results at screening
33. Participation in a clinical research trial within 30 days prior to randomization
34. Individuals who are cognitively impaired and/or who are unable to give informed consent
35. Any other condition which in the Qualified Investigator's opinion may adversely affect the individual's ability to complete the study or its measures or which may pose significant risk to the individual
36. Medical or psychological condition that in the Qualified Investigator’s opinion could interfere with study participation

5.3 Concomitant Medications

Participants who are currently taking any prescribed medications must agree to maintain their current method and dosing regimen during the course of the study unless recommended by their physician.

Use of over-the-counter medication or natural health products that affect glucose metabolism is prohibited within 2 weeks of randomization and during this study, these include:

Natural Health Products containing:

- Cinnamon (but culinary use is permitted)
- Fenugreek (but culinary use is permitted)
- Chromium
- High dose Magnesium
- Ginseng (*Panax ginseng*)
- Bitter melon (*Momordica charantia*)

Over-the-Counter medications containing:

- Decongestants (i.e. ephedrine, pseudoephedrine)

5.4 Early Withdrawal

Personal reasons

As stated in the Informed Consent Form, a participant may withdraw from the study for any reason at any time.

Removal by Qualified Investigator:

Participant discontinuation should be considered at the discretion of the Qualified Investigator. The circumstances of any discontinuation have to be documented in detail in the participant file and final report. If possible, the evaluations planned for the end of study will be carried out at the time when the participant is withdrawn from the study. A participant leaving the study prematurely will NOT be replaced by another. It is understood by all concerned that an excessive rate of withdrawals can render the study un-interpretable; therefore, unnecessary withdrawal of participants should be avoided.

Criteria for removal of participants from the study will include:

Clinical reasons

A participant may be withdrawn from the study if, in the opinion of the Qualified Investigator, it is not in the participant's best interest to continue. Any participant who experiences a serious adverse event (SAE) may be withdrawn from the trial at the discretion of the Qualified Investigator. A participant will also be withdrawn due to adverse events causing clinically significant illness or the need for prohibited medication(s) during the trial. Any female participant who becomes pregnant during the course of the trial will be withdrawn.

Protocol violation

Any participant found to have entered this study in violation of the protocol will be discontinued from the study at the discretion of the Qualified Investigator. This will include any participant found to have been inappropriately enrolled (did not meet eligibility criteria). Participant non-compliance includes not showing up for study visits, not taking the investigational product as directed, or refusing to undergo study visit procedures. Participants who are found to be taking prohibited medications or supplements without the knowledge of the Qualified Investigator will also be withdrawn. Any major protocol deviations (i.e., those that increase the risk to participants and/or compromise the integrity of the study or its results) will result in participant discontinuation.

6 INVESTIGATIONAL PRODUCT

6.1 Manufacturing and Storage

The investigational product will be provided to KGK by the Sponsor. The investigational product will be carefully stored at the study site in a lockable, limited access area, accessible only to study team personnel in compliance with pertinent regulations. Only authorized persons will have access to the investigational product. The products will be stored at room temperature and will not be exposed to direct sunlight or heat. The investigational products will be kept in a locked investigational product storage room at KGK Synergize Inc. on receipt. An accountability log will be kept for the investigational products.

All unused investigational product will be returned to the study sponsor by KGK (at the sponsor's expense) or destroyed on receipt of written confirmation from the sponsor at study closeout (within one month of last participant visit).

Manufactured by:

Allen Flavors Inc,

23 Progress Street

Edison, NJ 08820

6.2 Labeling and Coding

The investigational product will be labeled according to the requirements of ICH-GCP guidelines and applicable local regulatory guidelines. Investigational product will be randomized and coded by an unblinded person at KGK who is not involved in data collection or analysis.

6.3 Investigational Product (Good Idea^{TM})

Dietary Ingredient	Quantity (Qty)
L-Leucine	xxx mg
L-Threonine	yyy mg
L-Lysine	zzz mg
L-Isoleucine	uuu mg
L-Valine	uuu mg
Chromium picolinate	250 µg

Non-medical ingredients: Carbonated water, citric acid, lemon natural flavor, sodium benzoate, potassium sorbate

6.4 Placebo:

Ingredients: Carbonated water, citric acid, lemon natural flavor, sodium benzoate, potassium sorbate

6.5 Directions for investigational product preparation

One serving of Good Idea^{TM} is 12 fl. oz. and will be portioned out for participants. However, as one bottle of Good Idea^{TM} contains 10 fl. oz. the extra 2 onces of Good Idea^{TM} required for the serving size will be obtained from another bottle at each time of dispensing.

6.6 Directions to be followed prior to visit 2 and visit 3:

Test meals: Bread will be provided frozen at screening, all bread will be purchased from one and the same batch and will consist of white wheat bread made from refined wheat flour only and not contain any milk or other added fibers or proteins, all bread should be frozen directly after purchase by the clinic site. 24 hours prior to visit 2 (V2) and 3 (V3), participants will be instructed to remove frozen bread from the freezer and left to thaw in a sealed plastic bag.

Ham will be packed in portions in aluminum foil and kept frozen until 24 hours prior to V2 and V3. The standardized sandwich should be prepared in the morning of every test day by clinic coordinators. Type and brand of bread, ham and butter utilized for the standardized test meals.

1. 24 hours prior to V2 and V3, participants should avoid physical exercise, alcohol, and high fibre foods (such as whole grain, beans and lentils).
2. Participants will be advised to drink water *ad-lib* from morning till 10pm on the day prior to their clinic day. At least eight 250 ml cups of water to ensure adequate hydration in preparation for blood sampling.
3. The evening meal before V2 and V3, participants are instructed to eat a low fiber dinner (white wheat pasta with any type of sauce) at approximately 6pm. Participants will be instructed to consume the identical low fibre dinner meal at approximately the same hour before each study occasion.
4. Between 9pm – 10pm, participants will be instructed to eat any number (minimum 1 and maximum 4) of white wheat bread slices with optional spread/toppings, and a free choice of drink. Participants will be instructed to record the number of slices of bread consumed prior to V2. Participants will be required to consume the identical number of slices of bread with the same spread and drink prior to V3.
5. After this meal, participants are required to commence fasting and not eat or drink anything before they are served the standardized test breakfast at the clinical trial center the following morning (12 hours fasting).

6. All participants will be required to consume 250ml of water at the time of wake up prior to visiting the clinic.
7. Participants are advised to write down exactly what and when they eat and drink during 24 hours before the first visit, so they can repeat the same behavior for the following visit

6.7 Directions to be followed day of visit 2 and visit 3:

- In the morning (between 9am and 10am) after 12 hours of fasting, eligible participants will arrive at the clinic and will be randomized.
- Participants will consume the test product (12 fl. oz. of Good Idea^{TM}) together with a standardized test meal or consume the placebo together with a standardized test meal. The standardized test breakfast is white wheat bread with butter and ham.
- Participants will be instructed to consume 175 ml on an empty stomach immediately prior to the standardized meal, but after the pre-dose blood sample has been obtained, and the rest of the product together with the test meal. 175 ml of product should be drunk during the first minute. Thereafter, the participant will wait until the timer shows 3 min, and then the first sandwich bite should be consumed.
- The meal and remainder of the product should be consumed by alternating eating and drinking, in an even pace, within 11 minutes from the first bite. The remainder of the product should be portioned in three 50 ml-doses that are initiated at 3, 7 and 11 min, respectively. Participants will start consumption of product / meal a few minutes apart within the time frame of 15 minutes to ensure the accuracy of timing of blood samples. Participants should have finished the meal at 14 min from the start of taking product. At 14 min the final 30 ml of product is taken as a conclusion of the entire meal.
- Timing for blood sampling will commence at first sip of Good Idea^{TM}, time 0. The timing of the product consumption as well as the blood draw will be recorded where the timing of all subsequent blood draws will reference the product consumption.
- Participants will washout for a minimum of 7 days prior to the second test.

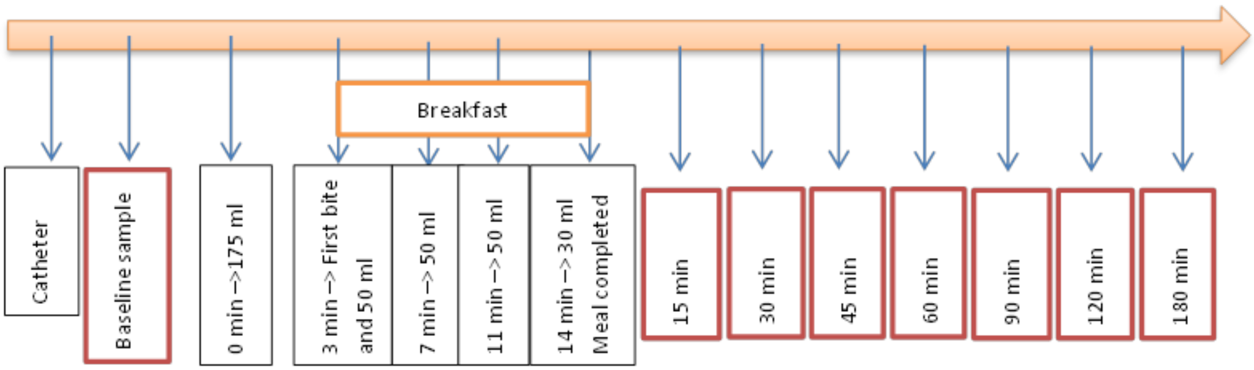

Figure 1. Schematic view of study day outline. Black boxes correspond to volume of carbonated drink (12 oz/355 ml) and red boxes mark time points where blood samples are drawn by the study nurse. Timing for blood sampling will commence at first sip of Good Idea^{TM}, time 0.

6.8 Randomization

A randomization schedule will be created and provided to the Investigator indicating the order of randomization. Each participant will be assigned a randomization code according to the order of the randomization list generated using www.randomization.com. Enrolled participants will be randomized to the different study arms at Day 0.

6.9 Unblinding and Allocation Concealment

Unblinding should not occur except in the case of emergency situations. In the event that a serious adverse event occurs, for which the identity of the investigational product administered is necessary to manage the participant's condition, the investigational product received by the participant will be unblinded and the investigational product identified. Concealment of the allocation of investigational product will be employed through the use of opaque sealed envelopes, each labeled with a randomization number. Each envelope will contain information regarding the investigational product associated with each randomization number. These envelopes will be readily available for the investigator to open in the event that it becomes necessary to know which product a participant is taking for the sake of the participant health care. The sponsor must be notified of any unblinding within 24 hours. Details of participants who are unblinded during the study will be included in the Final Report.

7 STUDY ASSESSMENTS

See section 12.4 for the schedule of assessments and procedures.

7.1 Screening (day -28 to day -1; visit 1)

At screening, an informed consent form will be given to the potential volunteer. They will be required to read the information and be given the opportunity to seek more information if needed, or provided with the option of taking the consent form home to review prior to making their decision. If agreeable, the volunteer will sign the consent form and receive a duplicate of the signed copy. Once consent has been obtained, the screening visit will proceed. Each volunteer will be sequentially assigned a screening number to be entered in the screening and enrollment log. Participants will be required to come to the clinic in a fasted (12 hours) state.

Screening visit includes:

1. Reviewing of medical history, concomitant therapies, and current health status
2. Assessment of the inclusion and exclusion criteria

3. Conducting a urine pregnancy test for potential female participants that are not of child bearing potential
4. Weight and height measurements (BMI calculation)
5. Seated resting blood pressure and heart rate measurements
6. Fasting whole blood sample collection for hematology: hemoglobin, hematocrit, platelet count, RBC, red cell indices, RDW, WBC and differentials (neutrophils, lymphocytes, monocytes, eosinophils, basophils)
7. Fasting whole blood sample collection for clinical chemistries: ALT, AST, bilirubin, creatinine, electrolytes (Na, K, Cl), glucose, and HbA1c
8. Assessment of self-perceived general health as assessed by the general health questionnaire (Appendix 2: Self-Rated Heath Item)

The next visit will be scheduled for potentially eligible volunteers. Eligible volunteers will be given a 3-day food record and a study diary. The 3-day food record will be used to record all food and drink consumed for 24 hours prior to the next visit and two other days that week (3-day food record will consist of two weekdays and one weekend total), and participants will be instructed to attend the following visit in a fasted (12 hours) state. Participants will be dispensed a frozen loaf of white wheat bread and receive instructions on how to consume this as a standardized meal prior to their next visit. The study diary will be used to record the participant activity during 24 hours before their next visit.

7.2 Day 0 – Baseline (visit 2)

Eligible participants will return to the clinic, in a fasted (12 hours) state, for baseline assessments. Time of arrival at clinic to the pre-dose blood sample will be recorded to ensure consistency. Food and study diaries will be collected and reviewed. From this the time and quantity of standardized meals consumed the night before will be established for all participants.

Baseline assessment include:

1. Reviewing of concomitant therapies and current health status
2. Re-assessment of inclusion and exclusion criteria
3. Urine pregnancy test for potential female participants that are of child bearing potential
4. Randomizing eligible participants
5. All participants will be briefed about the procedure of the test meal and the time points for eating drinking and blood sampling.
6. Weight measurement (BMI calculation)
7. Seated resting blood pressure and heart rate measurements
8. Bio-impedance analysis to determine body composition
9. Pre-prandial (0 min) fasting blood samples will be collected by IV for glucose and insulin

10. Pre-prandial (0 min) fasting blood samples will be collected by a finger prick for glucose
11. The study product and a standardized meal will be provided and consumed in the directions outlined in sections 6.5 and 6.7.
12. Post-prandial blood samples will be collected by IV for glucose and insulin at 15, 30, 45, 60, 90, 120, and 180 minutes from start of test consumption. Timing for blood sampling will commence at first sip of Good Idea™, time 0.
13. Post-prandial blood samples will be collected by a finger prick for capillary glucose at 15, 30, 45, 60, 90, 120, and 180 minutes from start of product consumption. Timing for blood sampling will commence at first sip of Good Idea™, time 0.
14. Whole blood sample collection at end of visit for hematology: hemoglobin, hematocrit, platelet count, RBC, red cell indices, RDW, WBC and differentials (neutrophils, lymphocytes, monocytes, eosinophils, basophils) and clinical chemistries: ALT, AST, bilirubin, creatinine, electrolytes (Na, K, Cl), glucose, and HbA1c
15. Seated blood pressure and heart rate measurements at the end of the visit
16. Adverse events experienced during the clinic visit will be recorded
17. Dispensing of study diary and instructing participants on completion
18. Dispensing of 3-day food diary and instructing participants on completion
19. Participants will be required to arrive at clinic for V3 at the same time as V2

The next visit will be scheduled for day 8 (+6 days) at the same time as visit 2. Participants will receive instructions on how to consume the standardized meal prior to their next visit as per section 6.6. The food record dispensed will be used to record all food and drinks consumed for the 24 hours prior to their next visit and two other days. Participants will be instructed to record changes in concomitant therapies, any adverse events and their activities for the 24 hours prior to their next visit into their study diary. Participants will bring their completed study diary and completed 3-day food diaries to their next visit as well as, arrive to the next visit (day 8) in a fasted (12 hour) state. Participants will be reminded that they are required to come to the clinic at the same time as V2 for V3.

7.3 Washout period (7 days)

The next visit will be scheduled after a minimum of 7-day and maximum of 14-day washout period will be allowed to accommodate scheduling. During the washout period participants should maintain their current diet regimens and continue to abide by the study protocol as per the inclusion and exclusion criteria. Participants will be required to record their food consumption in 3-day food records. A phone call will be made to participants prior to V3 to remind them of the requirement for adhering to the meal plan.

7.4 Day 8 (+6; visit 3)

Participants will return to the clinic on day 8 (+6 days) in a fasted (12 hour) state for their final assessments. Food and study diaries will be collected and reviewed. The time and quantity of standardized meals consumed the night before will be established for all participants.

Day 8 assessment include:

1. Participants will be required to arrive at clinic for V3 at the same time as their V2 visit
2. Review of concomitant therapies and adverse events
3. All participants will be briefed about the procedure of the test meal and the time points for eating drinking and blood sampling
4. Weight measurement (BMI calculation)
5. Seated resting blood pressure and heart rate measurements
6. Bio-impedance analysis to determine body composition
7. Pre-prandial (0 min) fasting blood samples will be collected by IV for glucose and insulin
8. Pre-prandial (0 min) fasting blood samples will be collected by a finger prick for glucose
9. The study product and a standardized meal will be provided and consumed in the directions outlined in sections 6.5 and 6.7.
10. Post-prandial blood samples will be collected by IV for glucose and insulin at 15, 30, 45, 60, 90, 120, and 180 minutes from start of product consumption. Timing for blood sampling will commence at first sip of Good Idea™, time 0.
11. Post-prandial blood samples will be collected by a finger prick for capillary glucose at 15, 30, 45, 60, 90, 120, and 180 minutes from start of product consumption. Timing for blood sampling will commence at first sip of Good Idea™, time 0.
12. Whole blood sample collection at end of visit for hematology: hemoglobin, hematocrit, platelet count, RBC, red cell indices, RDW, WBC and differentials (neutrophils, lymphocytes, monocytes, eosinophils, basophils) and clinical chemistries: ALT, AST, bilirubin, creatinine, electrolytes (Na, K, Cl), glucose
13. Seated blood pressure and heart rate measurements at the end of the visit
14. Dispensing of follow-up study diary and instructing participants on completion

7.5 Clinical Assessments and Procedures

Calculations or measurements of specific parameters are required as indicated in the schedule of assessments. Instructions for determining these parameters are provided in the following sections.

7.5.1 Height, Weight

Weight measurements will be performed with shoes removed, and bladder empty. Participants will be weighed on the same scale at all visits.

At least two separate measurements will be taken at each visit. If the two measurements are more than 0.5 kg (1.1 lbs.) apart, a third measurement will be taken. Then the two closest values will be selected and entered in the database.

Measurement of height will be performed with the participant's shoes removed. The participant's knees will be straightened, and head held upright.

7.5.2 Blood Pressure

An in-office, seated resting blood pressure and heart rate will be determined from 3 measurements obtained at least 1 minute apart. One arm will be chosen and used consistently throughout the study. Blood pressure will be checked in both arms at the first examination. If a consistent inter-arm difference exists, the arm with the higher pressure will be used throughout the study. The arm selected for use at the initial visit will be documented in the study file.

The participant should be seated comfortably with the back supported and the upper arm bared without restrictive clothing. Feet should be flat on the floor, legs will not be crossed. The participant will rest in this position for at least 5 minutes prior to the first reading.

The same recording method and the same equipment will be used for each participant throughout the study.

7.5.3 Compliance

Investigational product compliance is not necessary in this study as the investigational products will be administered in clinic and participants will be asked to consume the product in front of clinic coordinators.

7.6 Laboratory Analyses

Blood samples will be drawn from the participants at screening (visit 1), day 0 (visit 2), and day 8 (visit 3) as indicated in the schedule of assessments (Appendix 1 Schedule of Assessments).

Protection of subject confidentiality will extend to all data generated from the assaying of these samples. These samples will be alphanumerically coded and the persons performing the analysis will not be aware of the subject's identity or the allocated product they received.

At screening (visit 1) whole blood will be collected in:

1. EDTA vacutainer tubes to generate plasma for:
    a. CBC analysis (1 tube)
    b. HbA1c analysis (1 tube)

2. SST vacutainer tube to generate serum for:
    a. Electrolyte (Na, K, Cl), creatinine, AST, ALT, and bilirubin, fasting glucose analysis (1 tube)

At day 0 (visit 2) whole blood will be collected in:

1. SST vacutainer tubes to generate serum for:
    a. Pre-Prandial Fasting Glucose and Fasting Insulin analysis (2 tubes)
    b. Post-Prandial Glucose and Insulin at 15, 30, 45, 60, 90, 120 and 180 minutes analysis (2 tubes per time point)
2. A drop of capillary blood will be collected by finger prick and analyzed by a blood glucose monitoring system for:
    a. Pre-Prandial blood draw for glucose analysis (0 min)
    b. Post-Prandial blood drawn for glucose analysis at 15, 30, 45, 60, 90, 120, 180 minutes

At day 8 (visit 3) whole blood will be collected in:

1. EDTA vacutainer tubes to generate plasma for:
    a. CBC analysis (1 tube)
2. SST vacutainer tube to generate serum for:
    a. Electrolyte (Na, K, Cl), creatinine, AST, ALT, and bilirubin, pre-prandial fasting glucose analysis (1 tube)
    b. Pre-Prandial Fasting Glucose and Fasting Insulin analysis (2 tubes)
    c. Post-Prandial Glucose and Insulin at 15, 30, 45, 60, 90, 120 and 180 minutes analysis (2 tubes per time point)
3. A drop of capillary blood will be collected by finger prick and analyzed by a blood glucose monitoring system for:
    a. Pre-Prandial blood draw for glucose analysis (0 min)
    b. Post-Prandial blood drawn for glucose analysis at 15, 30, 45, 60, 90, 120, 180 minutes

Life Labs or LabCorp central laboratory will be used in this study to measure blood parameters

Urine pregnancy test will be performed at the KGK Synergize clinic site.

7.7 Termination of the Trial

In the case of premature termination of the trial, participating investigators/participants, and the Institutional Review Board must be promptly informed of the termination.

7.8 Protocol Amendments

If amendments to the study protocol are required after approval such changes will be captured in writing the reasons for the change documented and signed and dated by the sponsor. Any such amendments may be subject to IRB and Health Canada review/approval prior to implementation. Exception: if it becomes necessary to alter the protocol to eliminate an immediate hazard to participants, an amendment may be implemented prior to IRB approval. In this circumstance, the

Investigator must notify IRB and Health Canada in writing within five (5) working days of the implementation.

8 Safety Instructions and Guidance

8.1 Adverse Events and Laboratory Abnormalities

8.1.1 Adverse Events

An adverse event (AE) is any untoward medical occurrence in a clinical investigation participant who has been administered an investigational product and which does not necessarily have a causal relationship with the investigational product. An AE can be any unfavorable and unintended sign (including an abnormal laboratory finding), symptom, or disease temporally associated with the use of a product, whether or not it is considered related to that product. Pre-existing conditions which worsen during a study are to be reported as AEs.

During the study, participants should record any adverse effects in their diary. At each visit the participant will be asked "Have you experienced any difficulties or problems since I saw you last"? Any adverse events (AEs) will be documented and in the study record and will be classified according to the description, duration, intensity, frequency, and outcome. The Qualified Investigator will assess any AEs and decide causality.

Intensity of AEs will be graded on a three-point scale (mild, moderate, severe) and reported in detail in the study record.

Mild: Awareness of event but easily tolerated

Moderate: Discomfort enough to cause some interference with usual activity

Severe: Inability to carry out usual activity

The causality relationship of investigational product to the adverse event will be assessed by the Qualified Investigator as either:

Most probable: There is a reasonable relationship between the investigational product and AEs. The event responds to withdrawal of investigational product (dechallenge) and recurs with rechallenge when clinically feasible.

Probable: There is a reasonable relationship between the investigational product and AEs. The event responds to dechallenge.

Possible: There is a reasonable relationship between the investigational product and AEs. Dechallenge information is lacking or unclear.

Unlikely: There is a temporal relationship to the investigational product administration but there is no reasonable causal relationship between the investigational product and the AEs.

Not related: No temporal relationship to the investigational product administration or there is a reasonable causal relationship between non-investigational product, concurrent disease or circumstance and the AEs.

8.1.2 Serious Adverse Event

A serious adverse event (SAE) is any experience that suggests a significant hazard, contraindication, side effect or precaution. It is any AE that results in any of the following outcomes:

Death

A life-threatening adverse event

Inpatient hospitalization or prolongation of existing hospitalization

A persistent or significant disability or incapacity

A congenital anomaly/birth defect in the offspring of a participant who received the study product

Important medical events that may not be immediately life-threatening or result in death or hospitalization but may jeopardize the participant or may require intervention to prevent one of the outcomes listed above. Examples of such events are intensive treatment in an emergency room or at home for allergic bronchospasm; blood dyscrasias or convulsions that do not result in hospitalization; or the development of drug dependency or drug abuse.

8.1.3 Unexpected Adverse Reaction

An unexpected adverse reaction is an adverse reaction, the nature and severity of which is not consistent with the applicable product information (e.g., Investigator's Brochure for an unapproved investigational product or package insert/summary of product characteristics for an approved product).

8.1.4 Laboratory Test Abnormalities

The investigator must assess the clinical significance of all abnormal laboratory values as defined by the compendium of normal values for the reference laboratory.

Any investigational product emergent abnormal laboratory result which is clinically significant, i.e., meeting one or more of the following conditions, should be recorded as a single diagnosis on the AEs form in the study record:

1. Accompanied by clinical symptoms
2. Leading to interruption or discontinuation of the investigational product

3. Requiring a change in concomitant therapy

This applies to any protocol and non-protocol specified laboratory result from tests performed after the first dose of the investigational product, which falls outside the laboratory reference range and meets the clinical significance criteria for liver and kidney tests as well as for hematology and clinical chemistry.

This does not apply to any abnormal laboratory result which falls outside the laboratory reference range but which does not meet the clinical significance criteria or those which are a result of an AE which has already been reported.

Any laboratory result abnormality fulfilling the criteria for a serious adverse event (SAE) should be reported as such, in addition to being reported as an AE in the study record.

8.2 Treatment and Follow-up of AEs and Laboratory Abnormalities

8.2.1 Treatment and Follow-up of AEs

AEs, especially those for which the relationship to the investigational product is suspected, should be followed up until they have returned to baseline status or stabilized.

If after follow-up, return to baseline status or stabilization cannot be established, an explanation should be recorded in the study record.

8.2.2 Treatment and Follow-up of Laboratory Abnormalities

In the event of participant-initiated withdrawal or clinically significant unexplained abnormal laboratory test values, the participant will be withdrawn from the study arm and will remain in the study and be required to attend all remaining study visits as part of a safety arm.

8.3 Reporting of SAEs and Unexpected Adverse Reactions

The Qualified Investigator will be responsible for classification of an AE as an SAE within 24 hours of notification. Causality should be signed off by the Qualified Investigator prior to reporting to ethics and regulatory bodies. Notification of any serious adverse events must be made in writing to the study sponsor. The IRB will be notified of all SAEs and unexpected adverse reactions. All SAEs will be reported to the Therapeutics Products Directorate (TPD) in an expedited manner.

The sponsor must notify the TPD of all serious adverse reactions as follows:

> If it is neither fatal or life threatening, within 15 calendar days after the day on which the sponsor becomes aware of the information; and,
>
> if it is fatal or life threatening, must be reported as soon as possible, but not later than seven (7) days after the day on which the sponsor becomes aware of the information.

9 STATISTICAL EVALUATION

9.1 Determination of sample size

The planned sample size for this study is 30 participants crossing over (total of 60)

Power calculations were performed to determine the required sample size to provide 80% power at the 0.05 alpha level (that is, to have an 80% chance of obtaining $p \leq 0.05$ significance) when comparing the mean iAUC $_{(0\text{ - }120\text{ min})}$ between product and placebo.

With an estimated 20% attrition over the course of this study, 80% power and $p \leq 0.05$ when comparing product to placebo, if the product produces at least a 35.8 mmol/L/min decrease, over placebo, in iAUC $_{(0\text{ - }120\text{ min})}$ then a total of 60 participants are required to be enrolled.

9.2 Analysis Population

- The **Safety Population** will consist of all participants who received any amount of either product, and on whom any post-randomization safety information is available.
- The **Intent-to-Treat (ITT) Population** consists of all participants who received either product, and on whom any post-randomization efficacy information is available.
- The **Per Protocol (PP) Population** consists of all participants who consumed at least 80% of investigational product doses do not have any major protocol violations and complete all study visits and procedures connected with measurement of the primary variable.

9.3 Analysis Plan

An effectiveness analysis based on the intent-to-treat population and an efficacy analysis based on the per protocol population will be performed. Variables will be tested for normality and log-normality. Log-normally distributed variables will be analyzed in the logarithmic domain. Non-normal variables will be analyzed by appropriate non-parametric tests.

Incremental area under the curve (iAUC 0-180 min) will be determined for each participant at each study period. The iAUC will be calculated using the trapezoidal approximation. The maximum concentration (C_{max}) and the time to maximum concentration (T_{max}) value will be reported and compared for each study group.

For each numerical endpoint, a summary table will be prepared with a variety of summary statistics including mean, standard deviation, median, minimum value, and maximum value for each time point. The changes from baseline will also be provided and summarized similarly. For parameters requiring the logarithmic transformation, the summary statistics will be provided as non-transformed values. Mean values will be displayed as graphs, with a separate line for each product, and error bars indicating ± 1 SEM. Mean changes from baseline will be graphed similarly. At the end of the study, all raw study data will be provided to the Sponsor as a MS Excel formatted file.

Statistical tests:

Numerical efficacy endpoints will be formally tested for significance between groups by the repeated measures ANOVA test. Significant efficacy of the product, relative to comparator, will be inferred if the difference in means is significantly different from zero ($p \leq 0.05$). Numerical endpoints that are intractably non-normal will be assessed by a rank based test.

Probabilities ≤ 0.05 will be considered statistically significant. All statistical analysis will be completed using the R Statistical Software Package Version 3.2.1 (R Core Team, 2015) for Microsoft Windows.

9.3.1 Premature Discontinuation Description

For each premature discontinuation, the following parameters will be listed: participant number, dates of start and end of treatment, and the reason of premature discontinuation.

9.3.2 Safety

For adverse events, a descriptive analysis will be given. Adverse events will be presented in a frequency table by category and treatment. Furthermore, description, frequency, severity and causality will be reported for each adverse event.

Continuous safety parameters (e.g. hematology, clinical chemistry, heart rate and blood pressure) will be summarized using a table including mean, standard deviation, median, minimum value, and maximum value for each measurement point. The changes from baseline will also be summarized similarly.

9.4 Protocol Deviation Description

Protocol deviations will be listed in the final study report.

9.5 Protocol Amendments

Once the protocol has been approved by the IRB and Health Canada, any changes to the protocol must be documented in the form of an amendment. All amendments will be documented in the final study report.

10 DATA COLLECTION AND STORAGE

All data collection and record storage will be done in compliance with ICH GCP Guidelines and applicable local regulatory guidelines.

11 ETHICAL ASPECTS OF THE STUDY

This study will be conducted with the highest respect for the individual participants (i.e., participants) according to the protocol, the ethical principles that have their origin in the Declaration of Helsinki, and the ICH Harmonised Tripartite Guideline for GCP.

11.1 IRB Approval

KGK Synergize Inc. will supply relevant documents for submission to an IRB for the protocol's review and approval. The following must be submitted to the IRB: This protocol, a copy of the informed consent form, and, if applicable, volunteer recruitment materials and/or advertisements and other documents required by all applicable laws and regulations. The IRB's written approval of the protocol and volunteer informed consent must be obtained before commencement of the study. The IRB approval must refer to the study by exact protocol title, number, and version date; identify versions of other documents (e.g., informed consent form) reviewed; and state the approval date.

KGK must adhere to all requirements stipulated by the IRB. This may include notification to the IRB regarding protocol amendments, updates to the informed consent form, recruitment materials intended for viewing by volunteers, local safety reporting requirements and submission of the investigator's annual/final status report to the IRB.

11.2 Volunteer Information and Informed Consent

Written consent documents will embody the elements of informed consent as described in the declaration of Helsinki and the ICH Guidelines for GCP and will be in accordance with all applicable laws and regulations. The informed consent form describes the planned and permitted uses, transfers, and disclosures of the volunteer's personal and personal health information for purposes of conducting the study. The informed consent form further explains the nature of the study, its objectives, and potential risks and benefits, as well as the date informed consent is obtained. The informed consent form will detail the requirements of the volunteer and the fact that he or she is free to withdraw at any time without giving a reason and without prejudice to his or her further medical care.

11.3 Potential Risks and Procedures to Minimize Risk

All potential risks are disclosed to study participants prior to their participation. The potential risks associated with this study include venipuncture and the associated risks. Risks associated with venipuncture include pain, bruising, and infection at the site. Alcohol swabs and proper venipuncture procedure will be followed to minimize the risk of infection.

12 QUALITY ASSURANCE AND QUALITY CONTROL

12.1 Auditing

All material used in clinical studies are subjected to quality control. Quality assurance audits may be performed by the sponsor or any health authority during the course of the study or after its completion.

The Investigator agrees to comply with the sponsor and regulatory requirements in terms of auditing of the study. This includes access to the source documents for source data verification.

12.2 Monitoring

An initiation meeting will be conducted by the sponsor or an approved representative (CRO). At this meeting, the protocol and logistical aspects of the study will be reviewed with the Investigator and all study staff.

Source documents will be reviewed to ensure that all items have been completed and that the data provided are accurate and obtained in the manner specified in the protocol. The participant files will be reviewed to confirm that:

1. Informed consent was obtained and documented;
2. Enrolled participants fulfilled all inclusion criteria and did not meet any exclusion criteria;
3. AE/SAE reporting has been performed as applicable;
4. Study visits have been conducted as per protocol and information has been recorded in the appropriate place in the source document;
5. The study product is being stored correctly and an accurate record of its dispensation to the study participants is being maintained (accountability).

Incorrect, inappropriate, or illegible entries in the participant files will be returned to the Investigator or designee for correction. No data disclosing the identity of participants will leave the study center. The Investigator and any designees will maintain confidentiality of all participant records.

The Investigator will permit trial-related monitoring, audits, IRB/IEC review, and regulatory inspections and will allow direct access to source data and documents for these purposes.

12.3 Data Management

Data required for the analysis will be acquired from source documentation (including laboratory reports) and entered into a Microsoft Office Access database designed specifically for this study. All data points entered into the study database are source data verified.

High safety standards for the transfer and storage of study data are guaranteed by the use of technologies such as password protection, firewalls and periodic backup to protect stored data. Writing access to the system will be limited to authorized personnel.

All data is archived for a period not less than 25 years from the date of completion of the study in accordance with Health Canada regulatory requirements.

Reference List

1 Pena MJ, Rocha JC, Borges N: Amin Acids, Glucose Metabolism and Clinical Relevance for Phenylketonuria Management. Annals of Nutritional Disorders & Therapy 2015;2:1-5.

2 Kaats GR, Keith SC, Keith PL, Leckie RB, Perricone NV, Preuss HG: A combination of l-arabinose and chromium lowers circulating glucose and insulin levels after an acute oral sucrose challenge. Nutr J 2011;10:42.

3 Brand-Miller JC, Stockmann K, Atkinson F, Petocz P, Denyer G: Glycemic index, postprandial glycemia, and the shape of the curve in healthy subjects: analysis of a database of more than 1,000 foods. Am J Clin Nutr 2009;89:97-105.

4 Nilsson M, Holst JJ, Bjorck IM: Metabolic effects of amino acid mixtures and whey protein in healthy subjects: studies using glucose-equivalent drinks. Am J Clin Nutr 2007;85:996-1004.

5 Gunnerud UJ, Heinzle C, Holst JJ, Ostman EM, Bjorck IM: Effects of pre-meal drinks with protein and amino acids on glycemic and metabolic responses at a subsequent composite meal. PLoS One 2012;7:e44731.

6 Frauchiger MT, Wenk C, Colombani PC: Effects of acute chromium supplementation on postprandial metabolism in healthy young men. J Am Coll Nutr 2004;23:351-357.

APPENDICES

12.4 Appendix 1 Schedule of Assessments

	Screening (day -28 to -1; visit 1)	Day 1 (baseline; visit 2)	1-Week Washout	Day 8 +6 (visit 3)
Informed consent	X			
Review inclusion/exclusion criteria	X	X		
Review medical history	X			
Review concomitant therapies	X	X		X
Height*, weight, heart rate, blood pressure	X	X		X
Urine pregnancy test	X	X		
Randomization		X		
Laboratory tests:<br>hematology: hemoglobin, hematocrit, platelet count, RBC, red cell indices, RDW, WBC and differentials (neutrophils, lymphocytes, monocytes, eosinophils, basophils)<br>Clinical Chemistry: ALT, AST, bilirubin, creatinine, electrolytes (Na, K, Cl), glucose, and HbA1c*	X	X		X
Self-perceived general health questionnaire	X			
Blood Samples:<br>T= 0 (pre-dose), 15, 30, 45, 60, 90, 120, and 180 min for glucose (IV and finger prick) and insulin (IV) analysis		X		X
BIA		X		X
Investigational Product dispensed		X		X
Standardized meal		X		X
Study diary dispensed		X		
Study diary returned				X
Three-day food diary dispensed	X	X		
Three-day food diary returned		X		X
Compliance calculated		X		X
Adverse events		X		X

*only measured at visit 1

12.5 Appendix 2: Self-Rated Heath Item

In general, would you say your health is: (Please circle one)

Excellent	1
Very Good	2
Good	3
Fair	4
Poor	5

Scoring:

Score the number circled. If two consecutive numbers are circled, choose the higher number (worse health); if two non-consecutive numbers are circled, do not score. The score is the value of this single item only. A higher score indicates poorer health.

Note:

The Self-rated Health Item was tested on 1 129 participants with chronic disease. N = 51 for test-retest and the test-retest reliability was 0.92.

The Self-rated Health Item was used in the National Health Interview Survey. In a number of studies self-rated health has been found to be an excellent predictor of future health.

Reference

http://patienteducation.stanford.edu/research/generalhealth.html
