# Supplementary material for: A novel nutritional supplement containing amino acids and chromium decreases postprandial glucose response in a randomized, double-blind, placebo-controlled study
Source: PLoS One. 2020 Jun 24;15(6):e0234237. doi: 10.1371/journal.pone.0234237 (PMC7313729; doi:10.1371/journal.pone.0234237)
Supplement: Attachment — Submitted filename: Response to reviewers_final.docx [file pone.0234237.s005.docx]

**Reviewer #1:**

This manuscript is vastly improved since the previous round of peer review, and I commend the authors for taking on board most of the reviewers' suggestions. There is still one major issue which the authors have not addressed, and a few minor ones which have arisen since the last round.

Major issues:
- in my comment (6) in the previous round, I made a recommendation on how authors should report means and SDs in the descriptive tables, and group means and SEs in the inference tables, along with effects as between-group differences with their 95% confidence intervals and p-values. The authors have done most of this, but have not reported the 95% confidence intervals for the differences/effects; this is very important and should be done. Even where authors have decided to report effects in relative terms (e.g. 24.7% reduction in venous serum iAUC), a 95% confidence interval for this effect should be reported - although I would generally recommend that you stick to absolute differences in the results and perhaps keep relative differences to the discussion, because your analytical method does not estimate relative differences. Reporting SEs and SDs is not necessary in the abstract, but the 95% confidence intervals are crucial.
- the authors report some rank transformation technique to calculate p-values due to non-normality of 'values' (I'm assuming you mean outcome measures here). Normality of outcomes is not a requisite to report p-values from an ANOVA/regression; it is only the normality of residuals that is assumed.

**Answer:**

We apologize for the imprecision of our last response. The rank transformation had been performed due to non-normality of both the data and the residuals. Ranking data, as was performed in the analysis, is one of the options for handling this issue (1,2). But our statistician advised that presenting the 95% confidence intervals of the non-ranked transformed data with ranked p-values has limited statistical value.

However, we have found that some literature explicitly recommends against examining data and residuals for normality (3), especially since the F-test for sample sizes larger than 20 or 30 where there are equal sample sizes per study arm (which we have) is robust against moderate departures from the assumptions of normality and homogeneity of variances (4). Thus, running the same test without prior ranking is also a plausible option, which would ensure consistency with the confidence interval (i.e. linear mixed model with treatment as main effect, subject number as random effect controlling sequence and period instead of a ranked linear mixed model with treatment as main effect, subject number as random effect controlling sequence and period). As could be expected, the p-values and means derived from this analysis are slightly different from the ranked data, since they are least squared means that have been adjusted for subject random effect, sequence and period. Hence, we have adapted the manuscript accordingly in the text and Table 2.

(1) Conover W. J. in Lovric M. International Encyclopedia of Statistical Science: Springer 2011, p. 1184-1193
(2) Sawilowsky S. S. Nonparametric tests of interaction in experimental design. Review of Educational Research. 1990; 60: 91-126
(3) Gelman A, Hill J. Data analysis using regression and multilevel/hierarchical models: Cambridge university press 2006. P 45-46
(4) Cohen J. Statistical power analysis for the behavioral sciences. 2 edn: Lawrence Erlbaum Associates, Publishers 1988, p. 407-408

Other points:
Abstract
- If you choose to report the SEs in the abstract despite the comment above, please indicate what quantities are in the brackets (in addition to specifying the confidence intervals). Also, consider rephrasing all through to refer to 'differences' rather than 'reductions'/'decreases' in quantities. For example, you could say: "... the supplement resulted in decreased venous blood glucose compared to placebo, mean (SE) of 69.3 (7.2) versus 52.2 (6.4) respectively, a difference of 17.1 mmol/L (95%CI X to Y, p-value x.xxx)". Please report the exact p-values unless they are less than 0.001.

**Answer:**

The phrasing in the abstract has been revised according to the suggestions.

- the following phrase is completely inappropriate and should be removed: "... missed statistical significance". It implies that statistical significance is some magical target to be hit or missed which is not the case. You either find evidence of a difference, based on a statistically significant finding, or you don't find that evidence; but phraseology such as this suggesting that you aimed to find a difference but missed it is very poor statistical reporting.

**Answer:**

The phrase has been removed.

Methods
- p5 line 94: do you mean "... a total of 2.6g of five amino acids (L-leucine...

**Answer:**

We specify the meaning of 5AA in the introduction and thus think it is appropriate to use that abbreviation when we talk about the exact same blend. Consequently, we kept the text unchanged.

- p6, 'Outcome measures': the iAUC-0,120min could still be better described than has been done so far. Does it represent a change in plasma glucose concentration up to 120 minutes after ingestion of the meal/product? If so, describe it as such (or similar terms). Same for the other iAUCs.

**Answer:**

An extra sentence has been added for clarification.

- p8 second paragraph of 'statistical analysis' - there appears to be an incomplete sentence beginning "Although simple, the trapezoidal rule..."

**Answer:**

We cannot see that the sentence is incomplete but have tried to rephrase to make it more clear.

- p8 line 178: you mean "complete observations on 57 individuals", not complete datasets.

**Answer:**

The sentence has been rephrased to clarify.

- p8 line 180: the paragraph on page 9 from line 184 should be moved to the sentence that ends with "imputation." on line 180. After that, the next sentence should begin with p-values, not probabilities, which has a very different meaning in this context.

**Answer:**

The paragraphs were restructured according to the comment.

- p9 line 187 - see comment above about normality.

**Answer:**

Sentence was removed, since statistical method was revised according to Reviewers suggestion.

- Table 2 - see comment above about reporting differences with their 95% confidence intervals and p-values

**Answer:**

95% confidence intervals were included.

- p14 line 8 - see comment above about "missing statistical significance", which is a very serious and important point about statistical reporting.

**Answer:**

The sentences have been rephrased.

**Reviewer #2:**

The Authors have minimally responded to the Referee’s comments.
As no new data have been added in the revised version, the Referee again suggests the Authors to submit the manuscript as a rapid communication to a journal dealing with nutrition and related topics.

**Answer:**

The manuscript has been under Review for publication in PLOS one for a very long time after recommendation by the board of PLOS Med. We sincerely hope to qualify for publication now, a novel submission to a different journal would definitely be a non-preferred option. In addition, we believe that PLOSone is the right journal for this study since it allows wide availability to the scientific community in a research area that affects millions of individuals worldwide.

The Referee does not hide the disappoint due to the Authors’ refusal to report the amounts of amino acids present in the supplement, which the Authors declare to be protected by a patent.

**Answer:**

We understand the Reviewers disappointment in this matter; however, we hope that there is some understanding that scientific work and investment of many years needs to be protected from patent infringement. We are very happy to share material or information after publication of the manuscript with individual scientists/researchers upon request.

We thank the Reviewer for working on this manuscript.
